# Trustworthy artificial intelligence in radiation oncology: cross-industry lessons for development, validation, and deployment

**DOI:** 10.3389/fonc.2026.1912362

**Published:** 2026-07-23

**Authors:** Malinda Zhu, Lang Gou, Chi Zhang, Dandan Zheng

**Affiliations:** 1 Department of Radiation Oncology, University of Rochester, Rochester, NY, United States; 2 College of Chemistry, University of California, Berkeley, Berkeley, CA, United States; 3 School of Biological Sciences, University of Nebraska-Lincoln, Lincoln, NE, United States

**Keywords:** AI, clinical decision support, human-AI collaboration, radiation oncology, trustworthy AI, workflow integration

## Abstract

Artificial intelligence (AI) is rapidly expanding across the radiation oncology workflow, with applications spanning imaging, contouring, treatment planning, quality assurance, outcome prediction, workflow automation, and clinical decision support. Although technical progress has accelerated substantially, successful clinical translation remains inconsistent. Many of the challenges limiting implementation are not unique to radiation oncology and have previously emerged across healthcare and other high-stakes industries. In this narrative review, we examine radiation oncology AI through the broader lens of cross-industry AI development and deployment. We first summarize the current landscape of AI applications in radiation oncology and then analyze representative examples of successful and unsuccessful AI implementation from healthcare and other sectors. These experiences reveal recurring themes that strongly influence clinical AI success, including data representativeness, robust validation, workflow-centered design, human-AI collaboration, uncertainty management, bias mitigation, operational boundaries, and continuous performance monitoring. We discuss how these lessons apply directly to radiation oncology, where AI systems must function within complex clinical workflows involving imaging, planning, adaptive treatment, quality assurance, and longitudinal patient management. Emerging agentic and multimodal AI systems further amplify both opportunities and risks associated with deployment. Ultimately, the future impact of AI in radiation oncology will likely depend less on isolated algorithmic performance than on the development of trustworthy clinical AI ecosystems. Successful implementation will require rigorous validation, seamless workflow integration, human oversight, regulatory governance, and continuous adaptation. Lessons from healthcare and other industries suggest that the greatest and most durable clinical value may arise from AI systems that augment human expertise, cognitive workflows, and multidisciplinary decision-making rather than replace clinical decision-makers.

## Introduction

Artificial intelligence (AI) is increasingly reshaping modern radiation oncology. Rapid advances in machine learning, deep learning, multimodal data integration, large language models (LLMs), and computational infrastructure have enabled AI systems to participate in nearly every stage of the radiotherapy workflow. Current applications include image synthesis and enhancement, auto-segmentation, treatment planning optimization, outcome prediction, quality assurance (QA), workflow automation and orchestration, and clinical decision support (1).

The growing interest in radiation oncology AI reflects both technological opportunity and clinical necessity. Yet experience from other industries suggests that technical capability alone rarely guarantees successful adoption. Radiation oncology workflows are highly data-intensive, operationally complex, and increasingly dependent on integration across imaging, planning, delivery, adaptation, and longitudinal outcome assessment (2, 3). Simultaneously, escalating treatment complexity, workforce pressures, and demands for personalization continue to challenge conventional clinical paradigms. AI, therefore, offers the potential not only to automate repetitive tasks but also to augment decision-making, improve efficiency, enhance consistency, and ultimately enable more adaptive and biologically informed radiotherapy paradigms (4).

**Figure 1:**
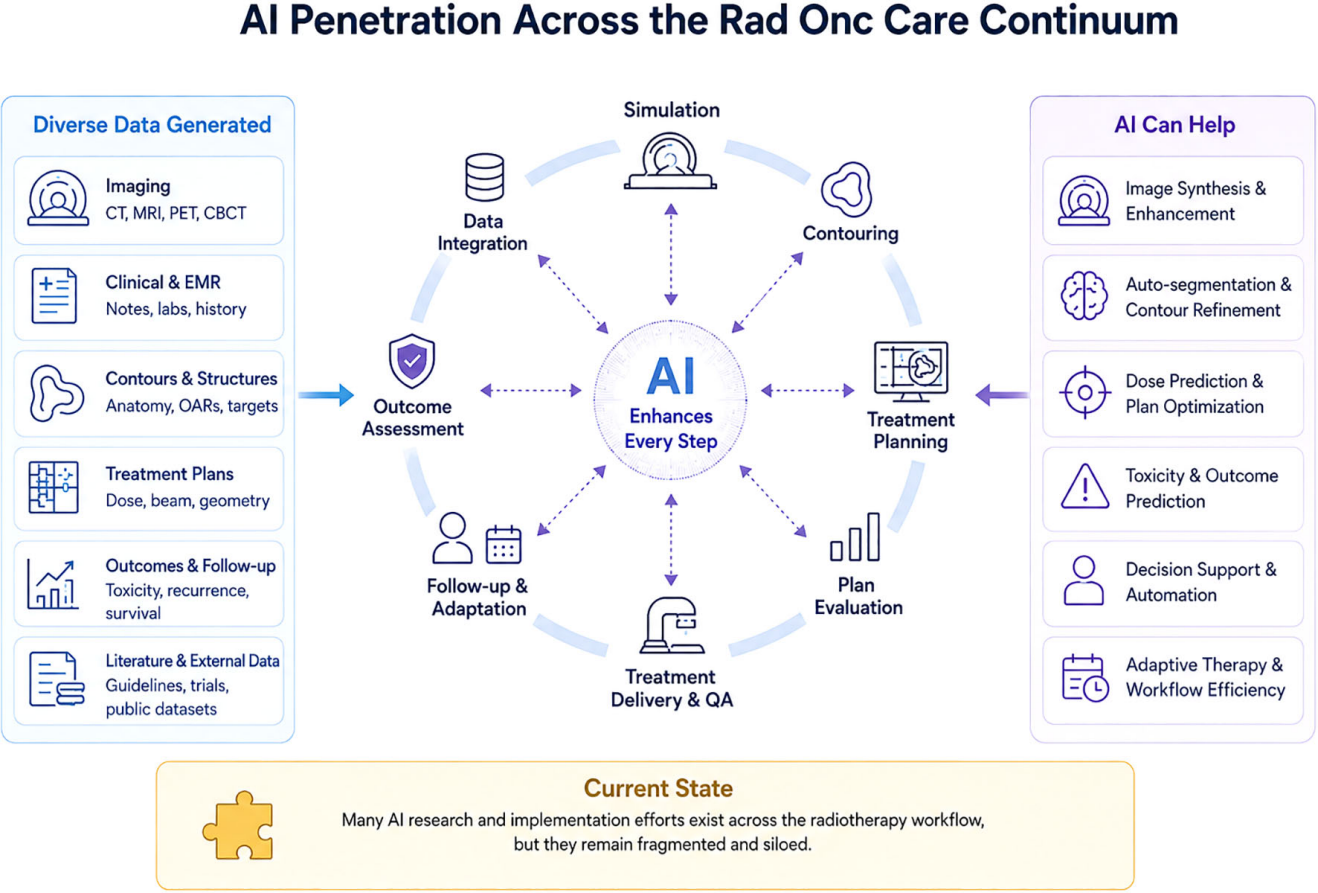
The broad penetration of AI across the radiation oncology care continuum. CBCT, cone beam computed tomography; CT, computed tomography; EMR, electronic medical record; MRI, magnetic resonance imaging; OAR, organ at risk; PET, positron emission tomography; QA, quality assurance.

**Figure 1** illustrates the broad penetration of AI across the radiation oncology care continuum, spanning imaging, contouring, treatment planning, treatment delivery, treatment adaptation, QA, and outcome assessment. Current applications already include synthetic imaging, auto-segmentation, dose prediction, toxicity modeling, adaptive planning, and workflow automation. However, despite substantial progress, many efforts remain fragmented and are variably integrated across the clinical workflow (5). This has also been a persistent disparity between AI innovations developed and validated at large academic centers and the broader adoption, implementation, and scalability of AI tools across diverse real-world clinical radiation oncology practices (6). Importantly, the central challenge facing radiation oncology is no longer whether AI can perform individual technical tasks. Rather, the emerging challenge is whether and how AI can be deployed safely, robustly, and meaningfully within real-world clinical ecosystems.

Much of the existing literature in radiation oncology AI focuses on model architectures, benchmark performance metrics, or disease-specific applications (5). Although valuable, these discussions often underemphasize broader translational issues, including workflow integration, human oversight, bias propagation, model drift, clinician trust, regulatory governance, and long-term adaptation. These are precisely the challenges that have already emerged across numerous AI-enabled industries.

Outside of radiation oncology, and indeed across medicine and other high-stakes industries, AI systems have experienced both transformative successes and highly visible failures (7). Transformative examples include AlphaFold, which revolutionized protein structure prediction, and Paige AI, which successfully translated foundation-model pathology into FDA-cleared clinical deployment (8, 9). In contrast, IBM Watson for Oncology struggled to deliver reliable treatment recommendations in real-world practice, while Amazon’s recruiting algorithm reproduced historical hiring biases embedded within its training data (10–12). Some platforms improved operational efficiency and clinical outcomes, whereas others collapsed under real-world complexity despite impressive developmental performance. These experiences provide a valuable opportunity for radiation oncology to learn from prior successes and mistakes rather than repeating them independently.

Unlike prior reviews that primarily catalog AI applications across individual stages of the radiation oncology workflow, this narrative review examines radiation oncology AI through the broader perspective of cross-industry AI development and deployment. Drawing on successes and failures from healthcare and other high-stakes industries, we identify recurring challenges involving data quality, workflow integration, human oversight, bias propagation, uncertainty management, regulatory governance, and long-term adaptation. We then synthesize these lessons into a practical framework for developing trustworthy clinical AI systems and increasingly integrated AI ecosystems.

This article is intended as a narrative review rather than a systematic evidence synthesis. Instead of comprehensively cataloging all AI applications in radiation oncology, we selected representative radiation oncology and cross-industry examples that illustrate recurring principles governing successful AI development, validation, implementation, and long-term deployment. Priority was given to influential publications, landmark implementation successes and failures, major regulatory developments, and examples with direct translational relevance to radiation oncology. Accordingly, this review emphasizes conceptual synthesis and practical implementation lessons rather than exhaustive literature retrieval or formal quality assessment of the selected AI examples.

## Current landscape of AI in radiation oncology

**Figure 2:**
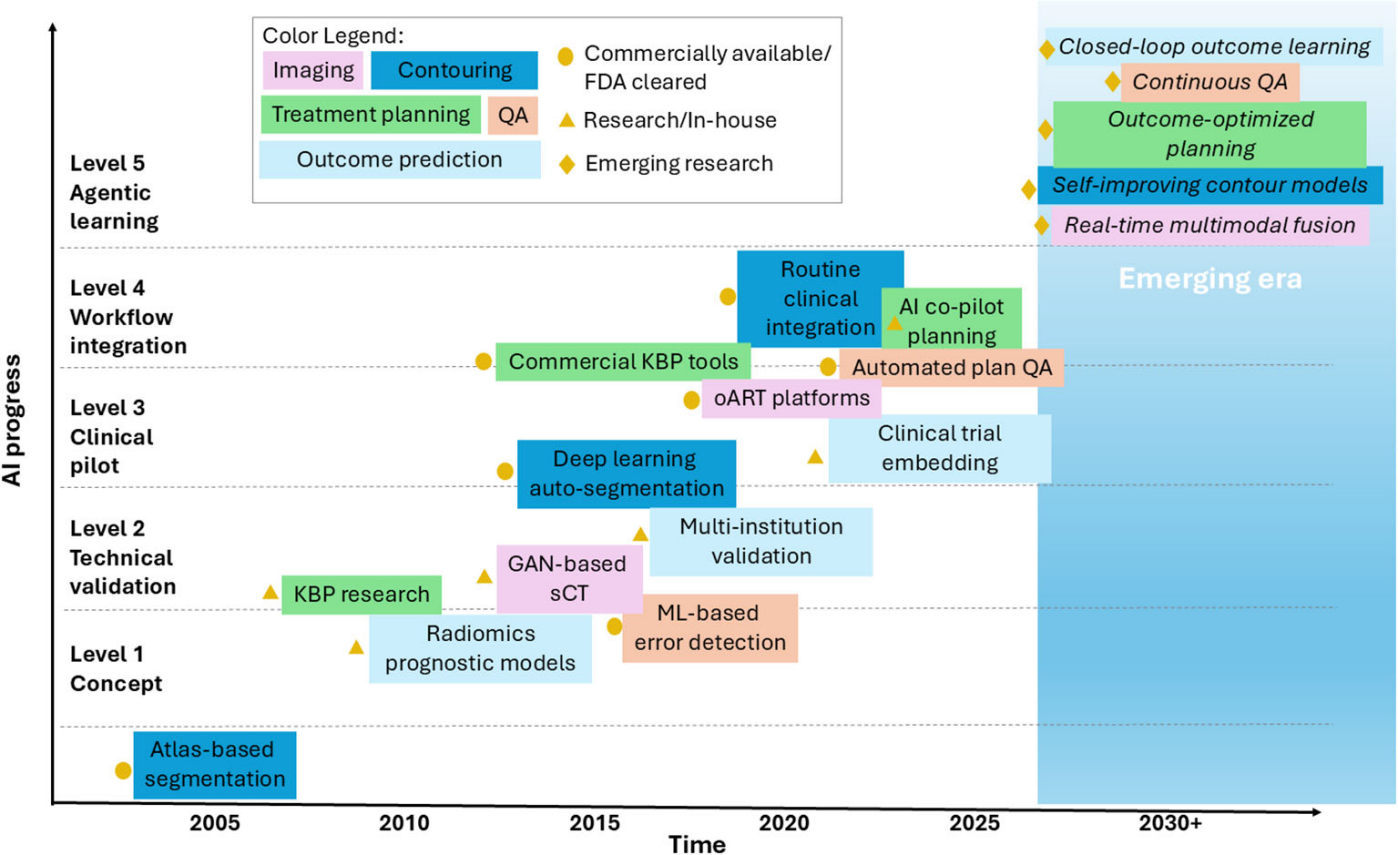
Current landscape of key AI applications in radiation oncology and the development timeline. GAN, generative adversarial network; KBP, knowledge-based planning; ML, machine learning; oART, online adaptive radiotherapy; QA, quality assurance; sCT, synthetic CT.

AI applications now span nearly every component of the radiotherapy workflow, although maturity and clinical adoption vary substantially across domains. **Figure 2** conceptualizes the evolving AI landscape in key radiation oncology applications according to developmental maturity and projected future integration. Regulatory maturity has progressed alongside technical development. Most commercially deployed radiation oncology AI systems are regulated as Software as a Medical Device (SaMD) and have entered clinical practice through FDA 510(k), *De Novo*, or similar international regulatory pathways. To date, the majority of cleared radiation oncology AI products focus on relatively bounded tasks such as auto-segmentation, image processing, treatment planning support, and quality assurance, reflecting areas where performance can be objectively validated and human oversight remains straightforward. Emerging FDA initiatives, including the Predetermined Change Control Plan framework for adaptive AI, may further facilitate safe deployment of continuously evolving clinical AI systems (13).

### Imaging

AI has become increasingly important in radiotherapy imaging workflows. Deep learning approaches have been applied to image denoising, artifact reduction, deformable image registration, image reconstruction, and synthetic image generation (14). In particular, synthetic computed tomography (sCT) generation from magnetic resonance (MR) imaging and cone beam CT (CBCT) has facilitated adaptive radiotherapy workflows and enabled streamlined online adaptive radiotherapy (oART) platforms (4, 15, 16). Despite promising early results, substantial challenges remain involving reproducibility, imaging standardization, generalizability, and biological interpretability. In the future, accurate and robust real-time multimodal image fusion or synthetic image generation would be desirable.

### Contouring

Auto-segmentation is one of the most clinically mature AI applications in radiation oncology (17). Deep learning-based contouring systems can substantially reduce contouring time and improve consistency for many organs at risk (OARs) and even for target structures (18, 19). Clinical deployment, overtaking previous atlas-based algorithms, has accelerated in recent years, particularly for relatively standardized disease sites. However, contouring performance remains highly dependent on anatomy, imaging quality, institutional contouring philosophy, and disease complexity. AI models may fail in cases of uncommon anatomy, low-quality imaging, or anatomically distorted cases (20). Furthermore, large spurious errors may still happen, such as generating contours in the wrong part of the anatomy or grossly wrong contours. Even seemingly small deviations in segmentation may carry clinically meaningful dosimetric implications (21). Consequently, auto-segmentation currently has varying degrees of penetration across different clinics (22). While it has been shown to improve clinical efficiency, it still requires careful review and often manual edits (23, 24). Nevertheless, auto-segmentation represents one of the clearest examples of successful clinical AI translation in radiation oncology, with several commercially available FDA-cleared platforms now routinely reducing contouring workload and improving workflow efficiency across many disease sites.

### Treatment planning

Machine learning and knowledge-based planning (KBP) systems have increasingly been incorporated into treatment planning workflows. These tools aim to improve planning consistency, reduce inter-planner variability, and accelerate optimization processes. Current approaches include automated rule implementation and reasoning, multicriteria optimization, dose-volume histogram (DVH) prediction models, dose prediction models, generative AI- or reinforcement learning-based planning, and AI-assisted agentic treatment planning (25). Although these systems may improve efficiency, they remain strongly influenced by the quality and characteristics of historical training data. Planning models frequently learn institutional planning preferences rather than universally optimal treatment strategies (26). As technologies and planning paradigms evolve, static planning models may also become increasingly vulnerable to performance drift. Furthermore, training these models requires significant expertise and time, and direct integration into commercial clinical treatment planning systems (TPS) is frequently unavailable. Consequently, wide clinical integration remains limited and relies on simple methods, while newer, more advanced methods typically remain in-house at large academic centers.

### Outcome prediction

Predictive AI models have been developed to estimate toxicity, local control, survival, treatment response, and other clinically relevant outcomes using combinations of imaging, dosimetric, genomic, and clinical variables (27). These approaches hold promise for individualized treatment adaptation and outcome-driven, biologically guided radiotherapy (28). However, outcome prediction remains challenging due to heterogeneous datasets, inconsistent endpoint definitions, evolving systemic therapies, and relatively limited sample sizes compared with many non-medical AI domains. Many published models remain retrospective, lack robust external validation, and demonstrate limited generalizability (27, 29). Consequently, their prospective evaluation in clinical trials remains highly limited.

### Quality assurance and workflow automation

AI applications in these areas are rapidly expanding. Proposed or investigated applications include machine QA and monitoring, patient-specific QA, automated plan checking, contour QA, delivery verification, continuous machine performance surveillance, incident learning, scheduling optimization, and treatment workflow orchestration (30, 31). Reported applications include EPID-based delivery verification, log-file analysis, machine performance prediction, automated image quality assessment, predictive maintenance, and anomaly detection using longitudinal machine QA data (31). Several of these applications have reached clinical implementation and illustrate that AI can augment not only planning workflows but also treatment delivery and equipment quality management (31). LLMs and natural language processing are also leveraged to further support documentation, communication, and clinical decision support (32, 33). A rapidly emerging application area involves physician-facing workflow support. Recent advances in LLMs have enabled ambient documentation systems to generate consultation notes, on-treatment visit documentation, treatment summaries, referral letters, and patient instructions directly from clinical encounters (34). Beyond documentation, these systems can assist with chart summarization, extraction of staging and treatment history from unstructured records, tumor board preparation, inbox management, and guideline retrieval (35, 36). For radiation oncologists, who routinely integrate pathology, imaging, multidisciplinary recommendations, prior therapies, dose-fractionation decisions, and longitudinal toxicity assessments, these tools may substantially reduce administrative burden and cognitive workload. Notably, documentation-focused AI represents a distinct paradigm from many traditional AI applications in radiation oncology, as its primary value lies in workflow augmentation rather than in autonomous clinical decision-making. Although these technologies promise to improve operational efficiency, widespread clinical integration remains limited. Furthermore, they introduce critical concerns, including automation fatigue, alert overload, hidden failure modes, and excessive reliance on AI outputs.

Collectively, these developments suggest that radiation oncology is transitioning from isolated AI tools toward increasingly interconnected and adaptive AI ecosystems. Yet the broader challenge remains not merely a matter of technical capability but of successful integration into complex human clinical systems.

## Cross-industry AI successes and failures as a framework for radiation oncology

As radiation oncology increasingly adopts AI, many of the emerging challenges resemble those already encountered in other industries. Cross-industry AI experiences, therefore, offer valuable insights into both successful deployment strategies and common failure modes. **Table 1** summarizes representative examples and their implications for radiation oncology.

**Table 1:** Cross-industry AI lessons and implications for AI in radiation oncology.

Lesson domain	Industry failure	Industry success	Radiation oncology parallel & R&D guidance
Data Integrity & Validation	IBM Watson for Oncology and Epic’s sepsis prediction model performed poorly in real-world settings because the models were trained on limited, retrospective, or nonrepresentative datasets, leading to unsafe recommendations and excessive false alarms (12, 37, 38).	Paige AI achieved FDA breakthrough designation through validation on large, diverse pathology datasets across multiple tissue types (8).	Auto-segmentation and planning models may fail on uncommon anatomy, evolving workflows, or institutional variations if these are absent from training data. Robust multicenter validation and inclusion of “edge-case” anatomy are essential to prevent clinically unusable “shelfware.”
Algorithmic Reasoning	Google Health COVID-19 models demonstrated “shortcut learning,” identifying confounding features (e.g., patient positioning) rather than true pathology (39, 40). LLM chatbots may generate medically plausible but unsafe oncology recommendations (41).	AlphaFold achieved robust protein structure prediction through biologically grounded model design, confidence estimation, and rigorous external validation against experimentally determined structures, helping ensure predictions reflected meaningful biological relationships rather than superficial correlations (9).	RT AI may learn physician-specific habits or geometric shortcuts rather than biologically meaningful relationships. Development should incorporate explainable AI, saliency mapping, counterfactual testing, and iterative expert review to ensure clinically meaningful reasoning rather than pattern mimicry.
Operational Boundaries	NYC’s “MyCity” chatbot provided inaccurate legal advice because it lacked mechanisms to recognize uncertainty or defer to authoritative sources (42).	Klarna implemented “trust thresholds,” requiring AI-to-human handoff when confidence falls below predefined limits (43).	AI deployment should incorporate explicit operational boundaries and uncertainty-triggered human intervention. Examples include mandatory physician review for high-risk anatomy, large PTV-OAR overlap, or low-confidence segmentation outputs.
Contextual Metrics	Zillow’s pricing algorithms failed despite high predictive accuracy because the models ignored contextual and subjective real-world factors (44).	Waymo evaluates autonomous driving systems using real-world safety performance, edge-case behavior, and intervention rates rather than isolated perception metrics alone (45).	Standard AI metrics such as Dice similarity coefficient and DVH indices may fail to capture clinically meaningful contouring errors or plan quality pitfalls. RT AI should incorporate context-aware endpoints, including dosimetric impact, toxicity relevance, functional status, and longitudinal clinical outcomes.
Integration & Infrastructure	Google Thailand’s diabetic retinopathy program failed partly because algorithms could not accommodate low-quality images and fragile local infrastructure (46). Volkswagen Cariad failed due to poorly integrated “black-box” software architecture (47).	Viz.ai improved stroke workflow efficiency by integrating imaging AI directly into clinical communication and logistical coordination pathways (48, 49).	AI tools requiring manual DICOM transfer, isolated workflows, or poor interoperability are unlikely to scale clinically. Successful RT AI should integrate natively with R & V and TPS while minimizing workflow friction.
Model Bias & Fairness	Amazon’s recruiting AI reproduced historical gender bias embedded within training data (11).	Paige AI reduced institutional bias by training foundation models on diverse pathology data spanning hundreds of institutions and dozens of countries (8).	RT AI trained on historical institutional data may perpetuate disparities in treatment selection, access, or planning philosophy. Diverse datasets, fairness auditing, and continuous retraining are critical.
Human Agency & Oversight	Overreliance on opaque automation systems contributed to workforce disruption, automation complacency, and silent system failures (50). Oncology chatbots have demonstrated partially non-concordant or potentially harmful treatment recommendations (41).	Paige AI and emerging ambient clinical documentation platforms have demonstrated successful human-AI collaboration by functioning as supervised amplifiers that augment expert performance while preserving clinician accountability (8, 51, 52).	AI should function as an assistive “agentic resident” or clinical copilot rather than an autonomous decision-maker. Successful deployment will likely emphasize workflow augmentation, information synthesis, documentation support, and decision assistance while preserving clinician supervisory authority, override capability, and active engagement.

DVH, dose volume histogram; FDA, Food and Drug Administration; LLM, large language model; NYC, New York City; OAR, organ at risk; PTV, planning target volume; R&D, research and development; R&V, record and verify system; RT, radiotherapy; TPS, treatment planning system.

### Data integrity and validation

One of the clearest lessons from prior AI deployment failures is that model performance is fundamentally constrained by the quality, diversity, and representativeness of the underlying training data. IBM Watson for Oncology became an early cautionary example after reports revealed clinically inappropriate and potentially unsafe treatment recommendations during deployment (12).

Similarly, Epic’s widely implemented sepsis prediction algorithm demonstrated poor sensitivity and excessive false-positive alerts when evaluated in independent, real-world clinical settings, despite promising retrospective performance during development (37, 38). This discrepancy occurred largely because the original model was trained on curated historical datasets that relied heavily on retrospective administrative billing codes rather than real-time clinical definitions of sepsis onset. When exposed to heterogeneous, fragmented, and noisy electronic health record data, it failed to replicate its development accuracy, triggering massive alert fatigue. In both cases, models trained on curated or retrospective datasets struggled when exposed to the messiness of live clinical workflows.

Conversely, successful systems achieved regulatory milestones by fundamentally shifting how clinical training data is sourced and scaled. Paige AI secured the first-ever FDA *De Novo* clearance for an AI tool in pathology (Paige Prostate) by moving away from traditional, manually annotated training sets, which are highly prone to local institutional bias (8). Instead, they built a massive foundation model trained on millions of unannotated whole-slide images sourced across hundreds of global institutions and dozens of countries. This cross-institutional diversity inherently accounted for variations in tissue staining, scanner types, and clinical populations.

Radiation oncology faces highly analogous dataset-driven challenges. Deep learning-based auto-segmentation and automated planning algorithms, trained predominantly on idealized anatomy, frequently degrade when confronted with the physical realities of the clinic. For example, auto-segmentation models can fail when they encounter anatomy unseen during training, such as postoperative changes, rectal spacers, abdominal compression, institutional contouring variability, or image artifacts (53, 54). For example, a head-and-neck auto-segmentation model trained predominantly on intact anatomy may perform poorly in patients with extensive postoperative reconstruction, bulky nodal disease, dental artifacts, or re-irradiation changes. Similarly, automated planning models developed using historical datasets may degrade when confronted with anatomy or clinical scenarios that were absent or underrepresented during training. Cases involving extensive postoperative reconstruction, metal implants, dental artifacts, hydrogel spacers, abdominal compression, severe anatomical distortion, re-irradiation, or rare anatomical presentations such as prune belly syndrome may represent out-of-distribution scenarios in which dose prediction and automated optimization may produce clinically unacceptable outputs, generating unrealistic objectives or clinically unacceptable plans despite excellent benchmark performance on standard datasets. Models that perform well under benchmark testing may become clinically unusable when exposed to these complexities of routine practice. Accordingly, ensuring the safety and generalizability of radiation oncology AI requires a shift toward multi-institutional development that prioritizes edge-case enrichment, rigorous external validation across disparate scanner types and acquisition protocols, and continuous, post-deployment performance auditing.

A critical gap in the radiation oncology AI literature is the limited use of prospective implementation studies that measure real-world clinical impact. Most published evidence remains retrospective and single-institutional, which risks optimistic performance estimates and limited generalizability (55). Future work should increasingly incorporate silent-mode validation, pragmatic clinical trials, cluster-randomized implementation studies, and standardized reporting of workflow outcomes, efficiency gains, and patient-centered endpoints. Demonstrating clinical utility under real-world conditions will be essential for sustainable adoption.

### Algorithmic reasoning and shortcut learning

AI systems can achieve exceptional predictive accuracy on paper while relying entirely on clinically irrelevant, confounding features. During the COVID-19 pandemic, multi-institutional studies revealed that many deep-learning models developed to detect pulmonary pathology on chest radiographs were derailed by “shortcut learning.” (39, 40) Instead of evaluating actual consolidated lung tissue, these computer vision models learned to exploit spurious correlations, such as the unique font styles of institutional text annotations, the presence of chest drains, or whether an image was captured portably in a supine position (highly correlated with severe disease) versus an outpatient upright scan. AI systems optimized strictly for a loss function will naturally exploit hidden mathematical shortcuts, which inevitably collapse under real-world distributional shifts (56). Similarly, contemporary LLMs can generate highly fluent, structurally flawless recommendations that are medically unsafe, masking severe omissions or pharmacological hallucinations behind an authoritative tone. For example, researchers found that 33.3% of the chatbot’s treatment recommendations were at least partially non-concordant with the National Comprehensive Cancer Network (NCCN) guidelines (41). It blended incorrect radiation and chemo advice with highly accurate text, making the errors nearly invisible to non-experts. These failures underscore the critical distinction between statistical correlation and clinically meaningful reasoning and warn against AI hallucinations. Conversely, successful AI systems often incorporate mechanisms that encourage learning of meaningful underlying relationships rather than superficial correlations. For example, AlphaFold achieved unprecedented protein structure prediction performance through biologically grounded model design, confidence estimation, and rigorous validation against experimentally determined structures, helping to ensure that predictions reflected fundamental structural biology rather than dataset-specific artifacts (9). Together, these examples illustrate that robust AI requires not only predictive accuracy but also evidence that model reasoning aligns with the underlying domain of interest.

Radiation oncology AI models face nearly identical systemic vulnerabilities. RT AI may learn physician-specific contouring habits, institution-specific planning conventions, or spurious correlations with imaging features such as immobilization devices, rather than clinically meaningful biological or anatomical relationships. In automated planning algorithm development or dose prediction, deep reinforcement learning or convolutional neural networks can easily memorize geometric regularities, standard couch angles, or a single institution’s non-protocol scripting habits rather than learning the underlying radiobiological and physical principles (57). Likewise, radiomic and clinical outcome prediction models are highly susceptible to historical treatment selection biases and confounding factors (58). A practical example is toxicity prediction. A model may identify feeding-tube placement or treatment breaks as powerful predictors of toxicity, not because they are biologically causal, but because they act as proxies for patients who already experienced severe treatment-related complications. Without careful causal reasoning, such models may achieve excellent predictive performance while offering limited guidance for treatment optimization. For instance, a model might misinterpret a clinician’s defensive decision to prescribe lower doses to a frail patient as a causal biological relationship between reduced dose and increased toxicity. Outcome models can also misinterpret planning trade-offs as biological phenomena. A model might flag a specific OAR DVH metric as ‘protective’ due to a negative correlation with toxicity, failing to realize this is a mathematical artifact of dose redistribution. The lower toxicity is actually driven by a reduction in a separate, more critical volume constraint, not a biological benefit inherent to the metric itself (59). To prevent these models from acting as mere superficial pattern matchers, future development must look beyond standard performance metrics and enforce explainable AI frameworks, such as saliency mapping to verify anatomical attention, counterfactual stress testing to evaluate model bounds, and iterative, expert-in-the-loop clinical validation.

### Operational boundaries and human oversight

Another recurring lesson from cross-industry AI failures is the danger of deploying systems without clearly defined operational limits. New York City’s “MyCity” chatbot, designed to answer questions about municipal regulations and public services, provided users with inaccurate and sometimes legally incorrect guidance on topics such as housing and employment regulations because it lacked reliable mechanisms to recognize uncertainty, verify information against authoritative sources, or escalate complex questions to human experts (42). In contrast, financial technology companies such as Klarna have implemented explicit “trust thresholds,” whereby AI systems can autonomously handle routine customer interactions but must transfer cases to human representatives whenever confidence falls below predefined thresholds or when requests involve ambiguity, exceptions, or potential financial risk (43).

Trustworthy clinical AI similarly requires transparent operational boundaries. Radiation oncology workflows contain numerous high-risk scenarios in which AI uncertainty may substantially increase patient risk, such as patients with unusual anatomy, postoperative changes, large overlap between targets and OARs, poor-quality or artifact-degraded imaging, and uncommon disease presentations that are underrepresented in training datasets. In such cases, inaccurate auto-segmentation, treatment planning, image registration, or outcome prediction may propagate errors directly into clinical decision-making and treatment delivery. Accordingly, AI systems should incorporate predefined escalation triggers, quantitative confidence estimation, and mandatory human review mechanisms whenever uncertainty exceeds acceptable thresholds. In practice, these triggers may include gross contour-volume outliers, unexpected target shifts during adaptive radiotherapy, unusually high predicted OAR doses, substantial disagreement between independent AI models, or major deviations from institutional planning objectives. Such scenarios should automatically prompt enhanced physician or physicist review rather than silent acceptance of AI outputs. Importantly, clinician trust is not established merely by high average performance metrics but by demonstrated robustness to rare but consequential failures, predictable behavior under uncertainty, transparent acknowledgment of limitations, and the ability to reliably identify situations that require human intervention.

### Contextual metrics and clinical relevance

Cross-industry experiences also demonstrate that conventional quantitative performance metrics may fail to capture real-world utility. For example, real estate company Zillow’s home valuation algorithms achieved strong predictive accuracy when assessed against historical sales data, yet the company’s home-buying initiative suffered substantial losses because the models inadequately accounted for local market dynamics, rapidly changing economic conditions, property-specific characteristics, and subjective factors influencing buyer behavior like “curb appeal” and local sentiment (44). Conversely, autonomous driving companies such as Waymo evaluate system performance using broader measures of real-world safety, including rare-event handling, intervention rates, and operational reliability, rather than relying solely on isolated perception or object-detection metrics (45). Together, these examples illustrate that technically impressive benchmark performance does not necessarily translate into successful real-world decision-making when important contextual factors are omitted.

Radiation oncology faces analogous challenges when evaluating AI performance with quantitative metrics that may not fully capture clinical impact. For example, the Dice similarity coefficient remains one of the most widely reported metrics in auto-segmentation studies, yet it may be insensitive to clinically meaningful errors (60). A segmentation of the optic chiasm, brachial plexus, cochlea, or other small structures may achieve a seemingly acceptable Dice score while still containing millimeter-level boundary deviations that substantially alter dose estimates (61). Likewise, two target contours may achieve high volumetric overlap despite clinically important differences in shape, extent, or inclusion of critical regions that could affect treatment coverage. Similarly, treatment-planning AI is often evaluated using limited DVH metrics or composite plan-score functions. For example, two lung SBRT plans may demonstrate nearly identical DVH statistics, yet differ substantially in chest-wall dose distribution, hotspot location, interplay robustness, or physician-perceived deliverability. These nuances are often immediately apparent to experienced planners but remain difficult to capture using conventional benchmark metrics. While useful, these metrics reduce complex 3D dose distributions to a limited set of numerical summaries and may fail to capture clinically relevant features such as dose spillage into adjacent tissues, hotspot location, dose conformity around critical structures, robustness to anatomical variation, or tradeoffs that experienced clinicians consider when judging plan quality. Furthermore, outcome prediction models may demonstrate strong discrimination, or calibration statistics yet provide little practical benefit if their predictions do not change treatment decisions, improve patient selection, reduce toxicity, or enhance clinical outcomes.

These limitations highlight the distinction between technical performance and clinical value. An AI model may outperform existing methods on benchmark datasets yet be neither clinically viable nor deliver measurable benefit to patients or clinicians in routine practice, either because it misses nuanced but important clinical considerations or because it is clinically viable but delivers little measurable benefit. Future evaluation frameworks should therefore prioritize clinically meaningful endpoints, including dosimetric consequences of AI-generated contours, efficiency gains across the treatment workflow, reduction in toxicity and treatment-related complications, improvements in adaptive decision-making, consistency across institutions and patient populations, and ultimately longitudinal patient outcomes. Such evaluation strategies more closely align AI assessment with the goals of clinical care rather than relying solely on surrogate technical metrics.

### Integration, infrastructure, and workflow compatibility

Many AI failures arise not from poor algorithms alone, but from inadequate integration into operational ecosystems. For example, Google Thailand’s diabetic retinopathy screening initiative developed an AI system capable of accurately detecting diabetic eye disease from retinal photographs, achieving 90% laboratory accuracy and reducing diagnostic confirmation time from 10 weeks to 10 minutes (46). However, for this AI tool created for those who lacked immediate access to eye specialists, real-world deployment proved challenging because images acquired in rural clinics were often lower quality than those used during development, necessitating repeated image acquisition and creating workflow bottlenecks. Limited internet connectivity and speed, staffing constraints, and mismatches between AI requirements and local clinical processes also reduced practical effectiveness despite strong technical performance. Similarly, Volkswagen’s Cariad software initiative sought to create a unified software platform across multiple vehicle brands and functions. Although individual components were technologically sophisticated, insufficient interoperability, integration complexity, and delays in coordinating software across existing vehicle systems contributed to major implementation challenges (47). Both examples illustrate that even highly capable AI systems may fail when they are not designed around the realities of operational infrastructure and end-user workflows.

In contrast, the stroke detection platform Viz.ai achieved clinical impact not merely through accurate image interpretation but by embedding AI directly into existing stroke-care pathways (48, 49). When the system identified a suspected large-vessel occlusion on imaging, it automatically alerted the appropriate specialists, shared relevant information with care teams, and accelerated coordination across emergency, radiology, and neurointerventional services. By reducing communication delays rather than simply generating predictions, the platform translated algorithmic performance into measurable clinical benefit. The closest analogue in radiation oncology may not be a contouring model or planning algorithm, but future AI systems that orchestrate adaptive workflows by automatically coordinating image review, contour approval, plan generation, QA, and treatment delivery.

Radiation oncology similarly depends on highly interconnected workflows involving imaging acquisition, contouring, treatment planning, plan review, QA, treatment delivery, adaptive replanning, and longitudinal follow-up. These processes span TPSs, oncology information systems, imaging platforms, delivery systems, and multidisciplinary clinical teams. An AI contouring tool that requires manual DICOM export and import, a planning model that operates outside the treatment planning environment, or an adaptive workflow that requires numerous additional user interactions may create friction that outweighs its theoretical benefits. The challenge is particularly relevant for adaptive radiotherapy, where the value of AI-generated contours or plan adaptation may disappear if piecemeal software solutions handle different workflow steps and require cumbersome data export, import, and manual verification, thereby prolonging treatment sessions and increasing on-table time. Similar considerations apply to AI-assisted applicator reconstruction, auto-segmentation for image-guided brachytherapy, and automatic planning for intraoperative brachytherapy, where highly accurate algorithms may provide limited clinical benefit if they remain fragmented across multiple platforms rather than functioning within a seamless end-to-end workflow. In these settings, workflow efficiency, usability, and seamless integration become as important as algorithmic accuracy. Likewise, AI-generated recommendations that are not visible within existing clinical workspaces are less likely to influence decision-making. Successful deployment will therefore require seamless interoperability, native integration, automated data exchange, and minimal workflow disruption. In practice, proprietary software architectures and vendor-specific interfaces, especially in highly streamlined systems such as integrated adaptive radiotherapy platforms, remain major barriers to scalable third-party AI deployment despite algorithmic performance.

### Human agency, bias, and the future clinical role of AI

Perhaps the most important cross-industry lesson is that successful AI deployment augments rather than replaces human expertise. A widely cited example is Amazon’s experimental recruiting algorithm, which was trained using historical hiring data (11). Because the underlying data reflected existing workforce imbalances, the system learned to systematically favor characteristics associated with previously hired candidates and penalize some indicators associated with female applicants. The issue was not malicious programming, but rather the algorithm’s replication of biases embedded within historical data. Similarly, studies evaluating LLM-based oncology chatbots have shown that while these systems can generate fluent, confident, and often helpful responses, they may also produce partially incorrect, outdated, or potentially harmful recommendations that are difficult for non-experts to recognize (41, 62). These examples highlight that AI systems can inherit limitations from their training data and may appear more reliable than they actually are. This concern is particularly relevant in oncology, where substantial disparities in cancer outcomes and survival already exist across racial, socioeconomic, geographic, and other underserved populations (63). AI systems trained on historical practice patterns risk perpetuating or even amplifying these inequities if fairness is not explicitly considered during development, validation, and deployment. For example, AI may learn disparities in referral patterns and access to advanced technologies such as SBRT, adaptive radiotherapy, MR-guided radiotherapy, or proton therapy. Without deliberate fairness evaluation, these systems risk reinforcing historical inequities under the appearance of objective decision-making.

Conversely, successful healthcare AI deployments have generally functioned as supervised decision-support systems rather than autonomous decision-makers. For example, Paige AI has been successfully deployed in digital pathology to assist pathologists by identifying suspicious regions, prioritizing cases, and reducing oversight errors, while leaving final interpretation and diagnosis under physician control (8). Similar patterns have emerged across healthcare, where AI has demonstrated value by supporting cognitive workflows, reducing the information-processing burden, synthesizing complex data, coordinating tasks, and supporting clinical decision-making rather than replacing expert judgment (64).

The rapid adoption of ambient documentation platforms and clinical AI copilots provides a contemporary example of this paradigm (51, 52). These systems generate draft documentation, summarize relevant clinical information, and streamline workflows, thereby reducing clerical burden while preserving physician accountability for clinical decisions. Their success suggests that healthcare AI may achieve broader acceptance when deployed as a cognitive amplifier rather than an autonomous clinician.

This framework may be particularly relevant to radiation oncology, where treatment decisions require continuous integration of imaging, pathology, treatment history, multidisciplinary recommendations, dosimetric tradeoffs, and longitudinal outcomes. Rather than functioning as an autonomous clinician, radiation oncology AI may be most effective as an assistive “agentic resident” that supports complex workflows while remaining under human supervision. Such systems could generate contours, propose treatment plans, identify anatomical changes during adaptive therapy, prioritize quality assurance tasks, summarize clinical information, or estimate toxicity risk. For example, an AI “agentic resident” might assemble prior radiation records, summarize cumulative dose constraints, identify candidate adaptive triggers, draft treatment recommendations, and prepare documentation before physician review. Importantly, such systems could synthesize information from current literature, institutional protocols, and individual physician practice patterns while integrating data distributed across years of clinical records, imaging studies, treatment courses, and multidisciplinary encounters. This capability may be particularly valuable in radiation oncology, where clinicians must routinely reconcile large volumes of longitudinal information and evolving evidence. By continuously aggregating and contextualizing data across patients and time, AI systems may help overcome inherent human cognitive limitations in information retrieval, pattern recognition, and longitudinal decision support while preserving clinician oversight and final accountability. In this model, AI reduces cognitive burden while final responsibility for treatment decisions remains with the clinical team. However, physicians, physicists, and dosimetrists must retain ultimate responsibility for contextual interpretation, safety oversight, and ethical judgment. In addition, clinicians must remain vigilant to ensure that AI systems do not perpetuate or amplify disparities embedded within the datasets used for model development.

Preserving meaningful human engagement is therefore essential not only for patient safety but also for mitigating automation complacency, preventing progressive workflow deskilling, and detecting system failures that may otherwise go unnoticed when clinicians become overly reliant on automated outputs (50). Accordingly, the greatest impact of AI in radiation oncology may lie not in replacing expertise, but in augmenting it: redistributing cognitive workload, accelerating routine tasks, expanding access to specialized capabilities, and enhancing decision support while preserving human judgment, accountability, and professional responsibility.

## The path forward for AI in radiation oncology

The next phase of AI development in radiation oncology will likely move beyond isolated task automation toward increasingly integrated, adaptive, agentic, and multimodal clinical ecosystems. Future systems may increasingly integrate radiation oncology data into continuously learning platforms that support real-time clinical decision-making across contouring, treatment planning, quality assurance, adaptive radiotherapy, and clinical documentation. Advances in large language models, multimodal foundation models, reinforcement learning, and agentic AI may further enable coordinated workflow orchestration across these domains. However, these same capabilities may also introduce new failure modes. Foundation models and agentic systems can propagate hallucinations across interconnected workflows, exhibit prompt sensitivity, misuse external tools, or amplify errors through autonomous task delegation (65). Rigorous evaluation frameworks, sandbox testing environments, uncertainty estimation, and human-in-the-loop safeguards will therefore be essential before widespread clinical deployment. Prospective failure mode and effects analysis (FMEA) may provide a useful framework for systematically identifying AI-associated failure modes, evaluating their clinical consequences, and implementing mitigation strategies before routine clinical deployment (66).

Long-term clinical impact will depend less on isolated algorithmic sophistication than on the coordinated development of trustworthy clinical AI ecosystems. Lessons from healthcare, aviation, finance, and other AI-enabled industries suggest that successful deployment requires simultaneous progress across data quality, model robustness, workflow integration, human oversight, regulatory governance, and continuous performance monitoring (67). Failure in any one of these domains may limit clinical impact, even when algorithmic accuracy is high. Future AI development should therefore follow a multilayer roadmap that prioritizes technical excellence alongside operational feasibility, clinical adoption, safety, equity, and long-term sustainability. Sustainable adoption will also require demonstration of economic value through improvements in efficiency, resource utilization, access to care, or clinical outcomes sufficient to justify implementation, maintenance, and governance costs.

Regulatory and institutional governance will be central to this roadmap. In the United States, the FDA’s Predetermined Change Control Plan framework represents an important step toward regulating adaptive AI systems while maintaining safety and effectiveness (68). Similarly, the European Union AI Act introduces risk-based oversight requirements for healthcare AI systems (10). In radiation oncology, these external regulatory frameworks will likely need to be complemented by local governance structures, including AI oversight committees, predefined performance thresholds, audit trails, version control, incident reporting mechanisms, and routine drift monitoring (69, 70). Such infrastructure will be especially important for managing continuously learning systems whose behavior may evolve over time. Model performance may also drift following routine clinical changes unrelated to the AI itself. Examples include treatment planning system algorithm or beam model upgrades, implementation of new CT simulation or CBCT reconstruction protocols, introduction of new immobilization devices, adoption of online adaptive workflows, or shifts in patient populations. These changes may gradually alter the clinical data distribution and degrade AI performance despite unchanged model parameters. This underscores the need for continuous local performance monitoring, periodic revalidation, and version-aware governance. Trustworthy AI deployment ultimately depends on multidisciplinary collaboration rather than any single individual or profession. While governance structures should be tailored to local practice, successful implementation requires coordinated clinical, technical, operational, and organizational oversight. **Table 2** presents representative stakeholder roles that together support the safe, effective, and sustainable integration of AI into radiation oncology workflows.

**Table 2:** Example multidisciplinary roles supporting trustworthy AI deployment in radiation oncology.

Stakeholder	Primary role in trustworthy AI deployment
Radiation Oncologist	Clinically validate AI outputs, approve AI-assisted decisions, and monitor clinical outcomes.
Medical Physicist	Commission and validate AI tools, perform QA, monitor performance drift, and investigate failures.
Dosimetrist/Radiation Therapist	Verify AI-assisted planning and workflow outputs, identify operational issues, and provide user feedback.
Clinical Informatics/IT	Integrate AI with clinical systems, ensure interoperability, cybersecurity, version control, and data governance.
Department Leadership/AI Champion	Lead implementation, user education, change management, and resource planning.
AI Oversight Committee	Establish governance policies, oversee auditing and drift monitoring, define escalation pathways, and ensure regulatory compliance.

Representative examples only; responsibilities may vary across institutions.

Importantly, future AI systems should be designed not only to improve technical efficiency but also to enhance clinically meaningful outcomes, reduce disparities in access to high-quality radiotherapy, and support more personalized and biologically informed care. As radiotherapy increasingly incorporates online adaptation, functional imaging, biologically guided treatment strategies, and longitudinal response assessment, AI may serve as a coordinating infrastructure that synthesizes growing clinical complexity into actionable decision support.

Ultimately, the future role of AI in radiation oncology will likely be that of a collaborative clinical partner rather than a fully autonomous replacement for human expertise. The most successful systems may be those that preserve clinician agency, enhance multidisciplinary decision-making, improve workflow resilience, and augment human judgment while maintaining transparency, accountability, and patient-centered care.

**Figure 3:**
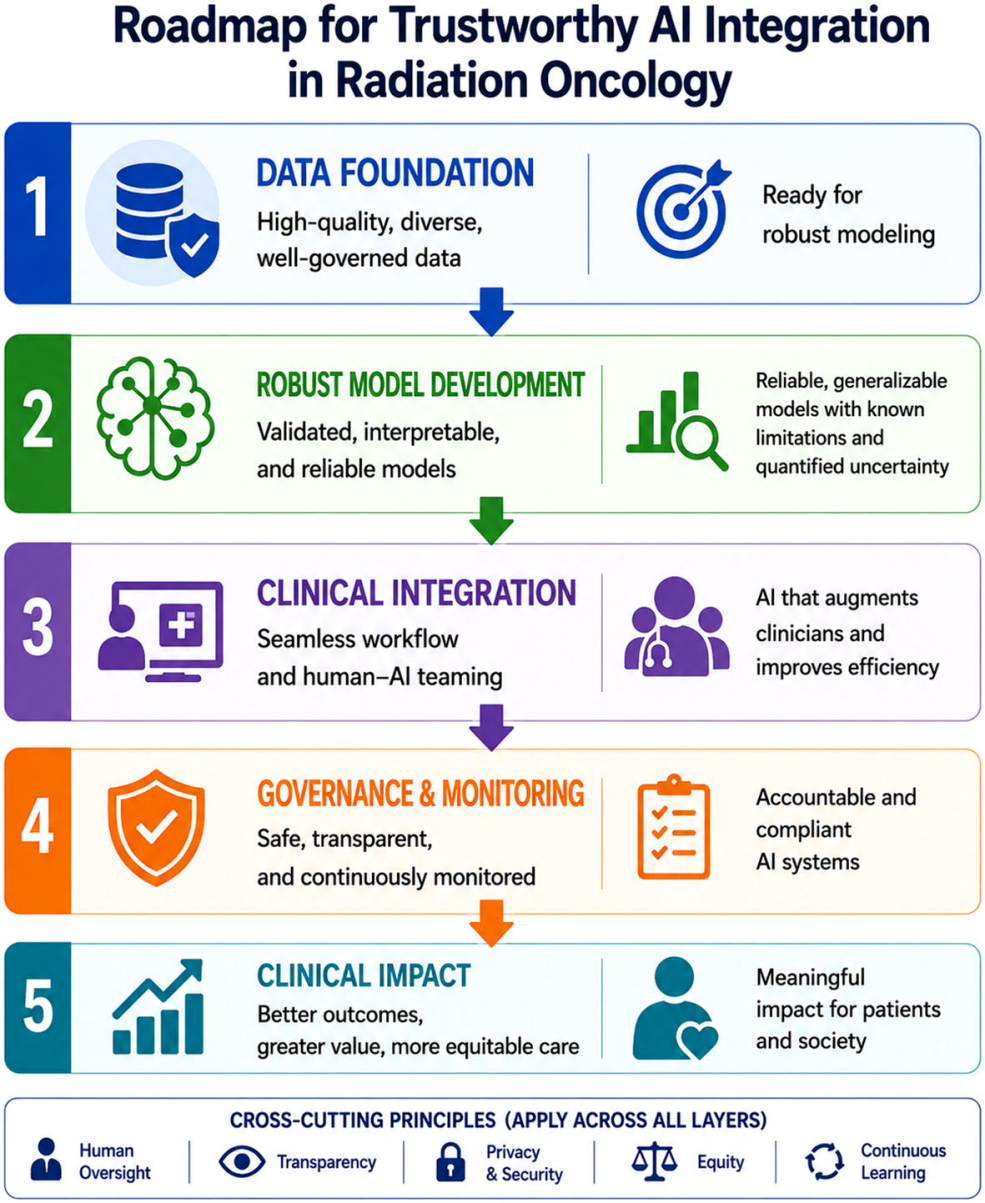
A roadmap for trustworthy AI integration in radiation oncology that illustrates a multilayer framework from data foundation to clinical impact.

A practical roadmap for this future is illustrated in **Figure 3**. Diverse multicenter datasets, standardized benchmarking, and bias mitigation form the foundation for robust AI development. These models must then undergo external validation, uncertainty quantification, and failure-mode analysis before clinical deployment. Successful translation will also require seamless integration, human oversight, confidence-triggered escalation, and interoperability with existing clinical systems. Finally, regulatory governance, continuous monitoring for model drift, and institutional oversight structures will be necessary to ensure safe long-term operation. Together, these interconnected layers provide a framework for achieving meaningful clinical impact while preserving safety, transparency, equity, and clinician accountability.

This review has several limitations. First, as a narrative review, it was designed to provide conceptual synthesis rather than a comprehensive systematic assessment of all published literature. Representative examples from radiation oncology and other high-stakes industries were intentionally selected to illustrate recurring implementation principles, and other relevant examples may also exist. Second, because AI is evolving rapidly, some technologies discussed, such as multimodal foundation models, clinical AI copilots, and agentic systems, remain early in clinical translation and should be interpreted as emerging future directions rather than established standards of care. Finally, the review emphasizes implementation science, governance, and translational considerations rather than detailed technical comparison of individual AI algorithms.

## Conclusion

Artificial intelligence is rapidly transforming radiation oncology, but experiences across healthcare and other high-stakes industries demonstrate that successful deployment depends on far more than algorithmic performance alone. The most durable AI successes have emerged from systems that combine robust validation, workflow integration, human oversight, and continuous monitoring, whereas many highly publicized failures resulted from nonrepresentative data, shortcut learning, workflow incompatibility, hidden bias, or inadequate governance. Importantly, some of the most impactful healthcare AI deployments have achieved value not through autonomous clinical decision-making, but through workflow augmentation, cognitive support, information synthesis, and reduction of administrative burden.

As radiation oncology evolves toward increasingly integrated clinical ecosystems, future development should prioritize prospective implementation, fairness auditing, uncertainty-aware deployment, and preservation of clinician supervisory authority. The goal is not autonomous replacement of clinicians but trustworthy AI that augments human expertise while improving efficiency, consistency, and patient care.

As radiation oncology enters an era of biologically guided, data-intensive, and increasingly adaptive treatment paradigms, the ultimate impact of AI will likely depend on how effectively the field integrates technological innovation with clinical judgment, multidisciplinary collaboration, and patient-centered care. By learning from prior cross-industry successes and failures, radiation oncology can move beyond viewing AI as a collection of isolated algorithms and instead build trustworthy clinical ecosystems that safely amplify human expertise, improve patient care, and realize the full potential of data-driven radiotherapy.
